# Surveying clinical trial participant satisfaction

**DOI:** 10.1186/1745-6215-16-S2-P45

**Published:** 2015-11-16

**Authors:** Christopher Bray

**Affiliations:** 1 Clinical Trial Service Unit & Epidemiological Studies Unit (CTSU), Nuffield Department of Population Health, University of Oxford, Oxford, UK

## Aim

This pilot study was conducted to evaluate participants’ satisfaction when attending appointments at one of the HPS2-THRIVE trial clinics, by collecting and comparing data about their expectations and perceptions.

## Methods

Using a modified version of the SERVQUAL questionnaire, participants were asked to assign a score to each of 10 service quality factors (SQFs) on a 5 point Likert scale to indicate (i) how important they considered each SQF in relation to their attendance at the clinic & (ii) their actual experience that day. Participants were also invited to describe anything they found particularly satisfying or dissatisfying about attending the study clinic.

## Results

44 (90%) of 49 consecutively attending participants agreed to complete this questionnaire, but 11 of the returned questionnaires were not fully completed. The remaining 33 questionnaires were included in a gap analysis. The overall mean score across all 10 SQFs was 4.6 for importance and 4.9 for actual experience. Actual experience scores exceeded importance for all SQFs except ‘Access’.

Free-text descriptions were provided on 23 questionnaires; descriptions of particularly satisfying experiences outnumbered those of dissatisfying experiences by 3:1. The main source of satisfaction related to staff friendliness and most of the dissatisfaction related to access.

## Conclusion

Overall, participants’ experience exceeded their expectations, the only exception being ‘Access’. ‘Friendliness’ was a key factor and should be included in the SQFs future. Nearly all participants were happy to complete the questionnaire and most were able to do so, but around 25% may have benefitted from assistance in completing it.

